# Black is beautiful (and protective): melanin synthesis in animals and plants

**DOI:** 10.1007/s00709-021-01693-3

**Published:** 2021-08-12

**Authors:** Peter Nick

**Affiliations:** Botanical Institute, Karlsruhe Institute of Technology, Karlsruhe, Germany

Melanin is a phenolic pigment that might qualify as one of the most political molecules ever. The reason for its impact on society has been a strange habit of certain members of *Homo sapiens*. The content of melanin in the human skin had been used over a long time to classify human beings into different categories, to which then, in consequence of melanin content, different degrees of human rights were assigned. While this practice, one of the manifestations of racism, is as bizarre as it appears, the idea that melanin content can be used for classification of *Homo sapiens* is still widespread. A good scientific response to this misconception has been given by the University of Jena in the so-called Jena Declaration (2019), commemorating the anniversary of Ernst Häckel (not only a prominent evolutionary biologist, but also a very active racist), who had died a century earlier. “The concept of race is the result of racism, not its prerequisite”. There is not much to be added to these clear and crisp words.

The biological background for differential melanin content in humans is linked with the selective pressure by UV irradiation during human evolution (Jablonski and Chaplin 2010). Melanin can absorb UV light and thus protects folate from photo-destruction; in the equatorial region, it helps to avoid skin damage. When modern humans moved out of Africa and migrated northwards, a second, antagonistic selective pressure appeared – to produce sufficient levels of vitamin D_3_, it is important that UV light can penetrate in the skin. In addition, at higher latitude, the danger of UV damage becomes irrelevant, such that a reduced accumulation of melanin was of selective advantage. There is no link whatsoever with any higher brain functions or mental patterns that had been inferred over centuries from the mere abundance of a UV-absorbing pigment in the skin.

It might help to understand the absurdity of this belief, considering that melanin is a pigment that can be found in all eukaryotic life forms and fulfils a couple of functions in addition to protecting against UV damage. Two contributions to the current issue highlight known but also unknown functions of melanin in human cells and in plants:

The first work by Skoniecka et al. (2021) investigates a specific type of skin cancers, so called amelanotic melanomes that are far more dangerous compared to their more common pigmented relatives. By comparing three cell lines of different provenience, the authors show that these cell lines can be induced to accumulate melanin, if supplemented with a sufficient level of tyrosine, the amino acid, from which the entire pathway initiates. The accumulated product is sequestered in specific compartments termed melanosomes, which already indicates a potential cytotoxicity. In fact, the precursors of melanin and phenolic compounds, such as the quinones, are known to exert damage. By over-saturating this sequestering, it should be possible to induce overflow into the cytoplasm, which should then result in the death of these cancer cells. In fact, the authors can show that induction of melanin can induce apoptosis, and they demonstrate this with a couple of markers such as externalisation of phosphatidyl serine, activation of caspases, and breakdown of mitochondrial electron transport. Interestingly, this cell death is not accompanied by oxidative burst, which might be linked with the strong antioxidant activity of melanin itself or its precursors. This work suggests new therapeutic lines to cure the dangerous “white” melanomes. By saturating their demand for tyrosine, it might become possible to get them channelled towards pigmentation, which should then culminate in self-elimination due to apoptotic cell death.

The second contribution, by Wolff Coutinho et al. (2021), puts the focus on plant melanin. Like their animal counterparts, phytomelanins provide protection, not only against UV light, but also to deposition of eggs by herbivorous insects. This type of pigments is usually overshadowed by its more prominent sisters, the anthocyanins, lending colour to flowers and fruits, sometimes also to foliage. The biochemical origin is similar, though. Both pigments derive from aromatic amino acids – while the desamination of phenylalanine into cinnamomic acid is the first committed step towards anthocyanin, phytomelanins derive from tyrosine. A classical model to study phytomelanins is the deeply pigmented seed coat of the Asparagales (Wittich and Graven 1995, 1998). However, many cellular details of these pigments have remained elusive, some even controversial. The authors have screened an extensive set of plant species to arrive at their experimental model, the Asteraceae *Piptocarpha axillaris*, which accumulates phytomelanins in the stem. The *terra incognita* already starts with the question, which cells are, in fact, responsible for the pigment that is found between the cells. Is it secreted? Or is it the remainder of cells that had accumulated phytomelanin internally and then collapsed, possibly by a mechanism resembling the apoptotic response of the melanoma cells mentioned above? The authors succeed in following the early genesis of this pigment and show that it is made in plastids in parenchymatic cells that differentiate into sklereids, lignified support cells that must undergo programmed cell death to fulfil their function. They also can reveal subcellular details of the process and test three concurrent models for the accumulation of melanins that have been developed to understand the subcellular deposition of tannins (phenolic plant compounds that derive from phenylalanine and, thus, can be used as model). They arrive at the conclusion that phytomelanin is formed in individual foci within the thylakoids that subsequently swell up, such that vesicles form that are interconnected like beads on a string. In contrast, the so called snail model can be discarded. While several questions to the genesis of phytomelanins have been solved, the question, how the pigment gets into the intercellular space, which is needed to do its job as protective surface, remains open.

Humans and plants represent life forms that are organised in a completely different manner. Nevertheless, both make use of phenolic compounds for pigmentation. The phenolic ring is endowed with three conjugated double bonds, establishing a delocalised orbital system which is volatile enough to be recruited for different functions, for instance, to donate an electron for a recipient molecule, or to accept the energy of a photon to produce absorption, i.e., colour. The first function seems to be the original task and shifts phenolic compounds, such as melanin, into the centre of redox homeostasis. The second function, colour, probably came in through the role of melanin as UV-absorbing pigment. That pigmentation became a signal (more evident in animals, less in plants, where this signalling function was rather adopted by the anthocyanins) is rather to be considered as a secondary “moonlighting” function of UV protection. Why such a strongly derived secondary function had been mis-used to classify humans into “races” becomes even more flimsy if one looks at it from such an evolutionary perspective.
